# Cognitive Diagnosis of Cultural and Rural Tourism Convergence

**DOI:** 10.1515/tnsci-2019-0004

**Published:** 2019-04-23

**Authors:** Yanjuan Liu

**Affiliations:** 1 School of Business Administration, Henan University of Animal Husbandry and Economy, Zhengzhou 450044, China

**Keywords:** Cognitive diagnosis, Adaptive neural network algorithm, Blockchain perspective, Cultural industry, Rural- tourism industry

## Abstract

Neural networks are widely used in the field of cognitive diagnosis. Cognitive diagnosis can diagnose the subjects’ knowledge of cognitive attributes according to their responses, so as to obtain the specific cognitive status of the subjects and provide remedial measures. The studies on the convergence of cultural industry and tourism industry are emerging, but the theoretical system needs to be improved. The research on the convergence mechanism between cultural industry and tourism industry can complement each other on the basis of independent theoretical system, which establishes relationship between the two theoretical systems. Based on the adaptive neural network algorithm and from the perspective of blockchain, this study takes cultural industry and rural tourism industry as examples to diagnose the industry convergence of rural cultural industry and rural tourism industry development, which will further consolidate the theoretical basis for the convergence and development of tourism industry and cultural industry, as well as contribute to promoting development of industry convergence.

## 1 Introduction

Blockchain is a new application mode of computer technology such as distributed data storage, point-to-point transmission, consensus mechanism, and encryption algorithm. Blockchain is an important concept of Bitcoin. It is essentially a decentralized database. At the same time, as the underlying technology of Bitcoin, it is a string of data generated by cryptography, and each block contains bitcoin network transaction information is used to verify the validity of its information (anti-counterfeiting) and generate the next block.

The original blockchain is a decentralized database that contains a list of blocks, with continuously growing and well-organized records. Each block contains a timestamp and a link to the previous block: the design blockchain makes the data unchanged— once recorded, the data in one block will be irreversible. Blockchain design is a protection measure, such as (applied) to a highly fault-tolerant distributed computing system. Blockchain makes hybrid consistency possible. This makes the blockchain suitable for recording events, headlines, medical records, and other activities that require data collection, identity management, transaction process management, and provenance management. Blockchain has enormous potential for financial disintermediation and has a huge impact on guiding global trade.

From the perspective of blockchain, the theoretical research on the convergence and development of cultural industry and tourism industry is beneficial to the government, enterprises and consumers. The research results are helpful for the government and the relevant departments to draw up policies of industry development, accelerate the optimization and upgrading of industrial structure, and promote the interactive development of industries. The results of the theory of industry convergence and development will also provide managers with more ideas of developing innovative products and elongate the chain of tourism industry and cultural industry. Consumers will get richer product choices and higher levels of spiritual and cultural needs ^[1]^.

Therefore, based on the adaptive neural network algorithm and from the perspective of blockchain, this study takes cultural industry and rural tourism industry as examples to analyze the industry convergence of rural cultural industry and rural tourism industry development, which will further consolidate the theoretical basis for the convergence and development of tourism industry and cultural industry, as well as contribute to promoting development of industry convergence.

## 2 Research on industrial integration development of rural tourism industry and cultural industry

The development of rural tourism must meet the needs of people’s life and entertainment, and the development of rural tourism should meet the needs of people’s life and entertainment, and follow the principle of sustainable development. Incorporating cultural and creative industries into rural tourism can, to a certain extent, strengthen the cultural connotation of rural tourism, so that the project can strengthen the cultural connotation of rural tourism to a certain extent and enrich the project. At the same time, it can also make some cultural resources of the village be effectively utilized, which provides a broad space for the development of rural tourism. Therefore, it provides a broad space for the development of rural tourism, and therefore can also achieve green and healthy development.

**Figure 1:**
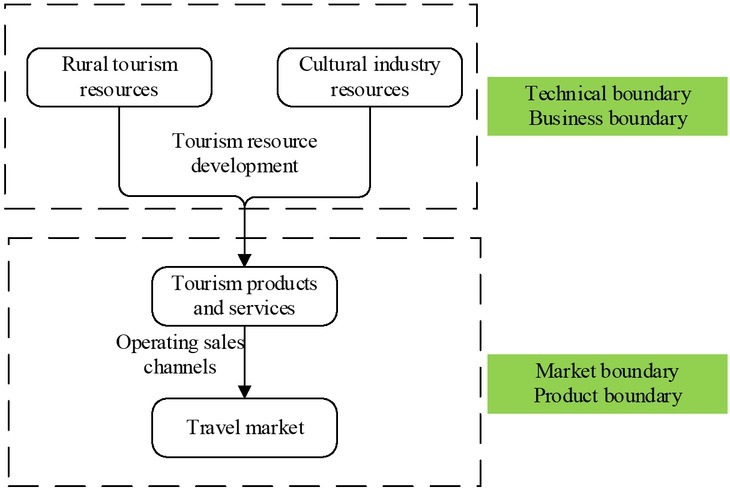
Industrial boundary of the rural tourism industry

The current studies involving the convergence and development of rural tourism industry and cultural industry are still in the embryonic stage. Industry convergence refers to the russification of industry boundary on the basis of technological convergence and digital convergence. Natural tourism resources and humanistic tourism resources in rural areas constitute the basis for the development of tourism industry. By certain production technological means, these tourism resources are transformed into tourism products and services that are put into the tourism market through operation means and sales channels ^[2]^. According to the classification principle of industry boundary, this study summarizes the industry boundary of rural tourism industry as shown in Figure 1.

**Figure 2:**
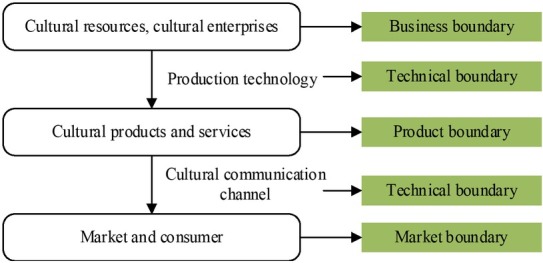
Industrial boundary of the cultural industry

Based on cultural resources, cultural industry development transforms cultural resources into cultural products and services that reach the cultural market through the cultural communication channels through certain production technological means according to the market demand. In this process, production technological means and cultural communication channels are the technological boundary of cultural industry. Cultural products and supporting services constitute the product boundary and the target market of cultural products becomes the market boundary while the enterprise, as the production and communication of cultural industry becomes the enterprise boundary of cultural industry (shown in Figure 2).

**Figure 3:**
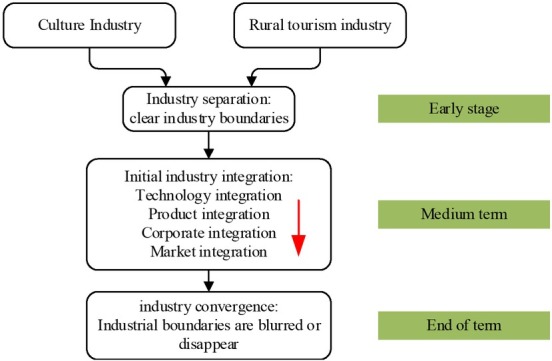
The process of integration of rural tourism industry and cultural industry

According to the theory of industry boundary, the shrinkage or disappearance of industry boundary is to adapt to the formation of new industry. The convergence of rural tourism industry and cultural industry is divided into three stages: earlier stage, middle stage and later stage. In the earlier stage, tourism industry and cultural industry are independent of each other, and each has its own industry boundary. They provide products and services with different characteristics and the fungibility between them is very small. Enterprise competition only occurs within the industry boundary. At this stage that is only the preparation stage, there is no real meaning of industry convergence. In the later stage, the industry boundary is vague or disappears, and the original value chain of the two major industries recombines into a brand-new value chain, forming a brand-new industry (as shown in Figure 3) ^3^.

## 3 The basic theory of adaptive neural network algorithm

The adaptive neural network algorithm synthesizes the basic idea of the artificial neural network. Through learning the information contained in the input-output data pair, it uses the sequential least square method and the error inverse propagation algorithm to adjust the parameters step by step, and finally achieves the goal of obtaining the parameters about the correct input-output relationship. This mainly includes two stages. The first stage is to use the improved genetic algorithm to search the optimal solution globally. The second stage is to use the optimal solution obtained in the genetic algorithm as the initial value of the neural network, and to use the neural network to search the local optimal solution. The second stage is still composed of three-layer neural networks, and the algorithm steps are similar to those of the standard neural network ^[4]^. The basic steps of the adaptive neural network algorithm are as follows:

### 3.1 Coding scheme

This mainly involves binary coding and real number encoding. The coding of the n^th^ neuron can be expressed by the real number encoding, as shown in Formula 1.

(1) $$\begin{array}{l} \alpha_{0}^{k},W_{11}^{k},W_{21}^{k},\text{L}\;\;\;\;W_{n1}^{k},V_{12}^{k},\text{L}\;\;\;\;V_{1q}^{k},b_{1}^{k},\text{L}\;\;\;\alpha_{i}^{k},W_{1i}^{k},W_{2i}^{k},\text{L L}\;\;\;W_{ni}^{k},V_{i2}^{k},\text{L}\;\;\;\;V_{iq}^{k},b_{i}^{k},\\ \text{L L}\;\alpha_{p}^{k},W_{1p}^{k},W_{2p}^{k},\text{L}\;\;\;\;W_{np}^{k},V_{1p}^{k},\text{L}\;\;\;\;V_{p2}^{k},b_{p}^{k},c_{1}^{k},c_{2}^{k},\text{L }c_{q}^{k} \end{array}$$

Where, $W_{1i}^{k},W_{2i}^{k},\text{L}\;\;\;W_{ni}^{k}$is the input connection weight of the corresponding neuron, $b_{i}^{k}$is threshold of hidden node, $V_{il}^{k},V_{i2}^{k},\text{L}\;\;\;\;\;\;{\text{V}}_{iq}^{k}$is the output weight of the corresponding neuron, $c_{1}^{k},c_{2}^{k},\text{L}\;\;\;\;\;c_{q}^{k}$is threshold of the output layer.

### 3.2 Group setting and initialization and determination of fitness function

In the population setting and initialization, M (≥2) subpopulations are randomly generated, where each subpopulation contains n solution vectors. The individuals in each population make the similarity between the individuals as small as possible according to a mode when all the individuals in each population can cover the whole solution as possible. In this way, the individuals in the population can keep the solution of each pattern as much as possible. The determination of the fitness function decides whether the network algorithm can find better results, which can determine the fitness function, as shown in Formula 2 ^[5]^.

(2) $$E\left(X\right)=\sum_{m=1}^{k}\sum_{j=1}^{q}{\left(Y_{mj}-{Y^{\prime }}_{mj}\right)}^{2},f\left(X\right)=\frac{1}{E+1}$$

Where, *Y_mj_* represents the expected value of the j^th^ output node of the n^th^ training sample; ${Y^{\prime }}_{mj}$is the actual value of the j^th^ output node of the n^th^ training sample.

### 3.3 Operation of network algorithms

In the adaptive neural network algorithm, fitness is the degree reflecting the fitness of the individual to the environment. Thus, so in the evolution, the average fitness and the maximum fitness of the population can be used to measure the condition of the current genetic operation, that is, if the average fitness of the next generation population is lower than that of the previous generation population, it shows that to a certain extent, the evolutionary operation of the algorithm generally evolves towards a method that is not conducive to the population, whereas the evolutionary operation is suitable for the development direction of the population ^[6]^. The adaptive genetic operator control formula is shown in

Formula 3:

(3) $$P=\left\{\begin{array}{c} P_{m}\times \left(1-P_{m}\right)\sigma \geq \xi \\ P_{m}\times \left(1+P_{m}\right)\sigma \leq \xi \end{array}\right\}$$

Where, *P_m_* indicates mutation probability; M is the number of iterations of the adaptive neural network algorithm; $\delta =\bar{f_{m-1}}-\bar{f_{m}},\bar{f}$expresses average fitness of population of genetic algorithm.

### 3.4 Flow chart of adaptive neural network algorithm

**Figure 4:**
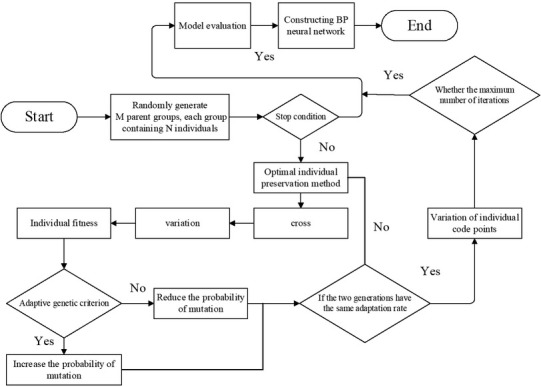
Flow chart of adaptive neural network algorithm

## 4 Taking cultural industry and rural tourism industry as an example, analyzing the influence of industrial integration on rural tourism industry from the perspective of blockchain-based on adaptive neural network algorithm

Although China’s tourism industry is still developing rapidly at this stage, the integration between cultural industry and tourism is not systematic. Many development models have been developed, such as market-oriented tourism creative models, tourism resource-oriented creative models, and innovative agricultural tourism models. We can learn from foreign tourism development models, such as from a consumer perspective, to conduct research on cultural characteristics and meeting people’s needs. From the perspective of supply and demand of cultural products, we will study the products and consumption of culture, and integrate the diversified industries to make tourism develop more rapidly.

If the industry wants rapid development, it needs to be combined with science and technology. By using science and technology to create more opportunities for development between industry and culture, it will promote the improvement of rural industries and related competitive advantages, thereby enhancing the competitiveness of rural tourism industry. By adding new products and concepts to the tourism industry, we can develop alternative tourism products by relying on science and technology, which enriches the cultural industry development form of rural tourism. The rural tourism has been continuously upgraded and extended. Therefore, technology can realize the development of rural culture.

In this study, the convergence of cultural industry and rural tourism industry in Enshi City of Hubei Province is taken as an example. The tourism number and tourism income are used as indexes to reflect rural tourism demand. An adaptive neural network prediction model with the help of neural network toolbox provided by MATLAB is established to predict rural tourism income and number of tourists in 2018-2021^[7]^.

### 4.1 Analysis of tourism demand in Enshi city

**Figure 5:**
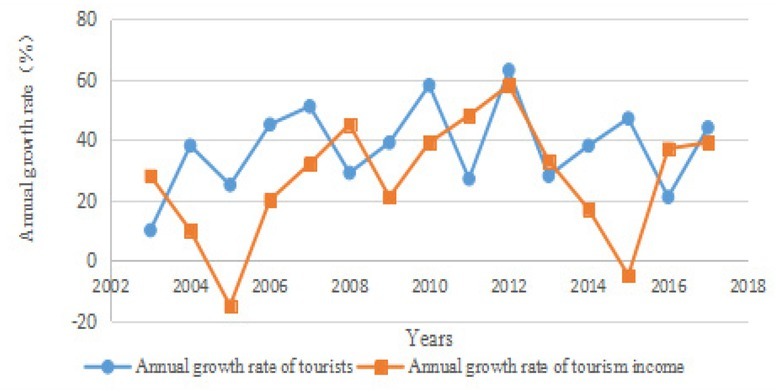
Annual growth chart of the number of tourists and tourism income in Enshi City from 2003 to 2017

For rural tourism, the material and intangible cultures such as cultural relics, human landscapes, production and life styles, folk customs and traditional festivals make rural tourism distinctive and dynamic, and the development of rural tourism promotes the inheritance and exchange of traditional national cultures in turn. Therefore, culture and rural tourism are inseparable, and complement each other ^[8]^. The number of tourists and tourism income is one of the three aspects that support the development of Enshi tourism. The basic situation is shown in Figure 5.

As shown in Figure 5, the number of tourists and tourism income in Enshi fluctuate in some years, but the overall development is very fast.

### 4.2 Multi-step prediction analysis of rural tourism number and tourism income in Enshi City based on adaptive neural network algorithm

According to the actual situation of data accumulation in Enshi City, the historical data of the number of tourists and tourism income in 2003-2017 are shown in Table 1 ^[9]^.

**Table 1:** Historical data on the number of tourists and tourism income in Enshi City from 2003 to 2017

Year	Amount of tourists(Unit: 10,000 people)	Tourism income(Unit: 10,000 yuans)
2003	3.50	25.26
2004	4.83	27.79
2005	6.04	23.62
2006	8.75	28.34
2007	13.22	37.41
2008	17.05	54.25
2009	23.70	65.64
2010	37.45	91.24
2011	47.56	135.03
2012	77.53	213.35
2013	99.24	283.75
2014	136.94	331.99
2015	201.31	315.39
2016	243.58	432.08
2017	350.76	600.60

**Figure 6:**
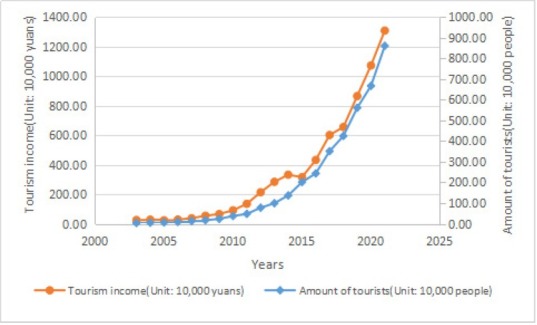
Forecast statistics of rural tourist population and tourism income in Enshi City

The tourism income of Enshi City in 2003-2017 is taken as the network input, and the predicted value of the rural tourism income in 2018 is calculated and output by the network. Similarly, after multi-step iteration, the rural tourism income in 2018-2021 is output, as shown in Figure 6.

According to the statistics of the number of tourists and rural tourism income in Enshi City (Figure 5), the number of tourists and tourism income in Enshi City will increase obviously in 2018-2021 if the number of tourists and tourism income develops as the dynamic trend in 20032017. According to the analysis of existing data, the reasons for the increase of number of tourists and tourism income in Enshi City are that Enshi City has always paid attention to the convergence of rural tourism and cultural industry, and Enshi has a good agricultural development trend. In the whole agricultural environment, the related agriculture, rural residents and rural areas are full of experience value. Consumers personally participate in these agricultural production links and rural atmosphere to experience agriculture. Thus, rural tourism with universal meaning comes into being, resulting in the increase of the number of tourists ^[10]^. In addition, Enshi increases investment in “food, accommodation, travel, purchase and entertainment”, and builds restaurants and accommodation areas with considerable quality and characteristics, as well as creates tourism commodities of unique style and entertainment areas of a rich variety so as to improve the quality of tourism services and open up tourism consumption space, which increases tourism income.

In order to verify the feasibility of the adaptive neural network model, a quadratic curve model and exponential model are established with the same training sample.

The simulation results of three models through simulation are obtained.

MAPE (mean absolute percent error %), R (correlation coefficient) and Z (reliability of output data %) are used to evaluate the accuracy of the model. The MAPE, R, Z values of the above models are obtained by calculation, as shown in Table 2.

**Table 2:** Comparison of the accuracy of different model simulation results

Predictive model	MAPE(%)	R	Z(%)
Adaptive neural network algorithm model	4.023	0.9999	100
Quadratic model	26.344	0.9943	64.23
Exponential curve model	22.359	0.9899	34.63

As can be seen from Table 2, the adaptive neural network algorithm model indicates that its simulation effect is better than that of other models from three parameters. Therefore, it is feasible to use the adaptive neural network algorithm to simulate the number of tourists and tourism income after the convergence of rural tourism industry and cultural industry in Enshi.

## 5 Conclusions

With the rapid development of social economy, people’s living standards have gradually improved, people’s demand for entertainment has become higher and higher, tourism has gradually become the main way of people’s life and entertainment, and the development of rural tourism in tourism has gradually started. Cultural creativity has been continuously improved in the context of economic development. By combining rural tourism and cultural creativity, new developments can be formed. This situation is in line with new economic trends. It provides abundant material resources and environmental protection for the development of rural tourism. Therefore, at this stage, we should pay more attention to the integration between the two, actively protect cultural resources and the environment, and make rural tourism develop faster.

Under the background of the structural reform of agricultural supply side, promoting the deep convergence of cultural industry and agricultural tourism industry plays an irreplaceable role in promoting the transformation and upgrading of cultural industry and agricultural tourism industry and enriching the local industrial structure. The adaptive neural network algorithm belongs to the data-driven model, which uses the nonlinear characteristic of the neural network to approximate a time series or the transformation of a time series. Through the clear logic relationship of the neural network, the value of the future time is expressed by the value of the past time. It is a complex and systematic project to study the development of rural tourism in Enshi City. This study hasn’t explored the convergence degree of rural tourism industry and cultural industry in Enshi City but simply uses adaptive neural network algorithm to analyze industry convergence from the perspective of blockchain. However, in reality, due to the limitation of the time series of tourism statistics, it is difficult to obtain enough historical data as training sample, affecting the accuracy of the prediction algorithm to a certain extent.
